# Catalysis Hub special collection: editorial

**DOI:** 10.1098/rsos.230484

**Published:** 2023-05-03

**Authors:** C. Richard A. Catlow

**Affiliations:** ^1^ Department of Chemistry, University College London, 20 Gordon St., London WC1 HOAJ, UK; ^2^ School of Chemistry, Cardiff University, Park Place, Cardiff CF10 3AT, UK

I am delighted to introduce a special collection of thematically related manuscripts in Royal Society Open Science. In 2021, I approached the journal to suggest commissioning a collection of papers derived from the work undertaken at member institutions of the UK Catalysis Hub. The Catalysis Hub is a consortium of over 45 institutions with research expertise in catalytic chemistry, led by the Universities of Cardiff, Bath and Manchester. The remit of the Catalysis Hub is to promote and coordinate catalytic science, including heterogeneous, homogeneous and biocatalysts, with a strong emphasis currently on predicting and optimizing new catalytic processes, developing catalytic science and technologies for achieving the circular economy, and catalysis for energy technologies and water purification.^1^ I have been involved with the Catalysis Hub since its foundation 10 years ago, but I have also been involved in a number of capacities with Royal Society Open Science at intervals since it was launched. As well as publishing papers in the journal, I was invited in 2018 by the then-Editor-in-Chief Prof. Jeremy F. M. Sanders to provide closing remarks for an event hosted at the Royal Society (of which I was Foreign Secretary) to launch the first ‘New Talent’ collection of chemistry papers.^2^

Following the success of the New Talent issue, it seemed timely to propose a special collection of catalytic chemistry papers derived from the Catalysis Hub community in a Royal Society journal, and I was pleased with the positive engagement both of the journal team and researchers at a number of the Catalysis Hub institutions in preparing this special collection. The collection is freely available at https://royalsocietypublishing.org/topic/special-collections/catalysis-hub. I am grateful that so many colleagues were willing to submit a paper to the collection, and the papers published represent positive responses to about 75% of the invitations initially issued. Many thanks to the contributors.

The papers in the collection explore catalytic chemistry and represent institutions in each of four constituent nations of the United Kingdom; they also showcase the global nature of science: several of the papers are international collaborations with non-UK partner institutions. As with the New Talent issue, many highlight the work of early career members of the community, and they illustrate the breadth of current catalytic science.

Ding *et al*. [1] examine the impact of palladium-doping of zeolites, in the aerobic oxidation of cinnamyl alcohol and benzyl alcohol. They find that dispersion of the catalytic active site is a key factor in optimizing activity.

In a review co-written with my colleagues Constantinos D. Zeinalipour-Yazdi, Justin S. J. Hargreaves and Said Laassiri [2], we reported on the recent work in ammonia synthesis probing mechanism via density functional theory. Importantly, we note that associative mechanisms for ammonia synthesis, which are closer to nitrogenase-mediated nitrogen-fixation, are becoming more promising and lower energy alternatives to dissociative mechanisms.

A third contribution to the collection demonstrates the possibilities available to catalytic chemists when borrowing from the natural world. In particular, it has been demonstrated that hard-to-synthesize cyclic peptides with a range of catalytic properties can be generated by using peptide cyclase 1 (PCY1) under comparatively mild reaction conditions [3].

Addressing some of the problems of our field (sustainability, environmental impact) is a review from Alice J. C. Wahart *et al*. [4]. This article surveys the advances in the use of oxidase enzymes to support more sustainable oxidation reactions. Not only does the use of oxidases in a range of industrially important processes have the potential to reduce the environmental footprint of these processes but also points to future opportunities for more selective chemistry available enzymatically catalysed reactions.

Maicon Delarmelina and I explore how the acid–base properties of zirconia—a widely used catalytic material—can be tuned by doping [5].

Catalytic chemistry will have a major part to play in the transition to Net Zero, but where the total removal of carbon from industrial processes is impossible, improving the efficiency of reactions and yield outputs are critical. The research reported by Tedstone *et al.* in their contribution to this collection is a good example of this: deploying novel catalytic methods to improve the efficiency of hydrocarbon cracking [6].

The orbital states and hybridization of furfural on a range of metal catalyst-doped surfaces are explored in the article of Chutia and colleagues [7].

Using a manganese oxide-based octahedral molecular sieve (OMS)-2 and Cu-doped OMS-2 allowed for a comparatively lower energy and comparatively efficient selective hydrogenation of levulinic acid into γ-valerolactone in a study by Nayan J. Mazumdar and colleagues [8].

Finally, Shuzhuang Sun and co-workers show how carbon dioxide capture can be integrated with its catalytic conversion to CO via the reverse water gas shift reaction—a crucial process in the development of sustainable fuels [9].

Taken together, it is hoped that the reader of this collection will not only gain a broader understanding of the current state-of-the-art of catalytic chemistry in general but also the wide range of fascinating work that is taking place in the UK Catalysis Hub. Furthermore, the collection demonstrates the immense value in collaboration at a range of scales in the scientific endeavour from the intra-institutional to the inter-institutional to the international.

I hope readers will find much to like in these papers; I have certainly enjoyed being a participant in the Catalysis Hub projects and have learnt much from my colleagues involved in this diverse and energetic field.

Finally, I would like to thank Andrew Dunn and the staff at the Royal Society for all their help in preparing this volume.

## Footnotes

1 https://ukcatalysishub.co.uk/ (accessed 5 April 2023).

2 https://royalsocietypublishing.org/topic/special-collections/new-talent (accessed 5 April 2023).
